# Ultrasensitive Wearable Pressure Sensors Based on Silver Nanowire-Coated Fabrics

**DOI:** 10.1186/s11671-020-03303-2

**Published:** 2020-03-30

**Authors:** Yunlu Lian, He Yu, Mingyuan Wang, Xiaonan Yang, Hefei Zhang

**Affiliations:** State Key Laboratory of Electronic Thin Films and Integrated Devices, School of Optoelectronic Science and Engineering, University of Electronic Science and Technology of China (UESTC), Chengdu, 610054 People’s Republic of China

**Keywords:** Pressure sensor, Silver-coated fabrics, High sensitivity

## Abstract

Flexible pressure sensors have attracted increasing attention due to their potential applications in wearable human health monitoring and care systems. Herein, we present a facile approach for fabricating all-textile-based piezoresistive pressure sensor with integrated Ag nanowire-coated fabrics. It fully takes advantage of the synergistic effect of the fiber/yarn/fabric multi-level contacts, leading to the ultrahigh sensitivity of 3.24 × 10^5^ kPa^−1^ at 0–10 kPa and 2.16 × 10^4^ kPa^−1^ at 10–100 kPa, respectively. Furthermore, the device achieved a fast response/relaxation time (32/24 ms) and a high stability (> 1000 loading/unloading cycles). Thus, such all-textile pressure sensor with high performance is expected to be applicable in the fields of smart cloths, activity monitoring, and healthcare device.

## Introduction

With the recent development of wearable electronics, there is an increasing demand for flexible pressure sensors in a multitude of applications including e-skin devices, health monitoring systems, and smart robots [1–8]. In order to be viably employed in these applications, pressure sensors must exhibit excellent sensitivity performance, thus, providing exhaustive information for accurate diagnosis or analysis.

To date, numerous methods have been developed to improve the sensor performance by optimizing the nanomaterials, including carbon nanotubes (CNTs) [1], graphene nanosheets [9], metal nanowires [10–19], conductive polymers [20], and their composite materials [21–26]. Particularly, Ag nanowire (AgNW) has been widely explored as the sensing materials or conductive fillers in pressure sensors because of its excellent electrical properties. For example, Wang et al. fabricated a flexible pressure sensor based on the AgNW-filled PU film, rendering a sensitivity of 5.54 kPa^−1^ at the pressure range of below 30 Pa [27]. Ho et al. reported a transparent crack-enhanced pressure sensor consisting of two laminated PDMS films bearing AgNW-embedded microfluidic channels [28]. However, most of these sensors were fabricated using airproof elastic substrates, which are unbreathable and uncomfortable to wear, thus limiting their practical applications.

More recently, textile-based pressure sensors have attracted increasing attention because of its softness, breathable, and biocompatibility, which makes it durable and wearable for long term. AgNWs have been widely used in the textile-based pressure sensors as sensitive layer. For textile-based sensors, a typical structure consists of flexible circuits covered with a conduction fabric, and they exploit a change in contact resistance between the circuits and the fabrics. When a pressure is applied, the two films contact, and a significant current is generated. For instance, Wei et al. demonstrated a wearable pressure sensor with the structure of two conductive AgNWs-coated cotton sheets [29]. Zhou et al. designed a pressure sensor with a printed textile electrode and AgNW-coated cotton fabric [30]. However, the pressure range is limited for the structure of these sensors. Thus, various structure designs have been proposed to improve the performance of pressure sensors. Zhong et al. developed an ultrasensitive piezoresistive sensor with high flexible, which is composed of POE nanofibers and AgNWs by a facile filtration method. The nanofibers are replicated on patterned nylon textiles with different fiber spacing [12]. Despite this progress, an all textile-based pressure sensor with ultrahigh sensitivity and structure design is seldom reported by far.

Here, we proposed a novel strategy for fabricating all-textile-based pressure sensors. The AgNWs solution was synthesized, and then the conductive fabric can be fabricated using a dip-coating method, which was done by dipping cotton pieces into the AgNW dispersion. The active sensing element contained double-layered AgNW-coated cottons with a cotton mesh spacer to secure the initial contact between them. The pressure sensing is based on changes in the electrical current due to the contact between the facing layers upon external pressure. This all-textile-based piezoresistive pressure sensor fully takes advantage of the synergistic effect of the fiber/yarn/fabric multi-level contacts, leading to the ultrahigh sensitivity of 3.24 × 10^5^ kPa^−1^ at 0–10 kPa and 2.16 × 10^4^ kPa^−1^ at 10–100 kPa, respectively. Meanwhile, the pressure sensor achieved a fast response/relaxation time (32/24 ms), and high stability (> 1000 loading/unloading cycles). Such devices have wide applications in smart clothes, activity monitoring, and healthcare device.

## Experimental Section

### Materials and Methods

The AgNWs solution was synthesized by hydrothermal method. First, the solution of PVP was added into EG; then, the mixture was stirring for 20 min to fabricate the solution of PVP/EG. Subsequently, the solutions of AgNO_3_/EG and NaCl/EG were prepared with a similar method. Second, the solutions of AgNO_3_/EG and NaCl/EG were added into PVP/EG, and the mixture was stirred and transferred into a reaction kettle. Third, the kettle was heated to 140 °C for 2 h and then to 160 °C for 30 min. Subsequently, the kettle was naturally cooled down to room temperature. The obtained precipitates were washed and centrifugal filtered with acetone and deionized water several times to form a white powder. Lastly, the obtained AgNWs were ultrasonically dispersed in ethanol.

### Fabrication of Pressure Sensor

**Fig. 1:**
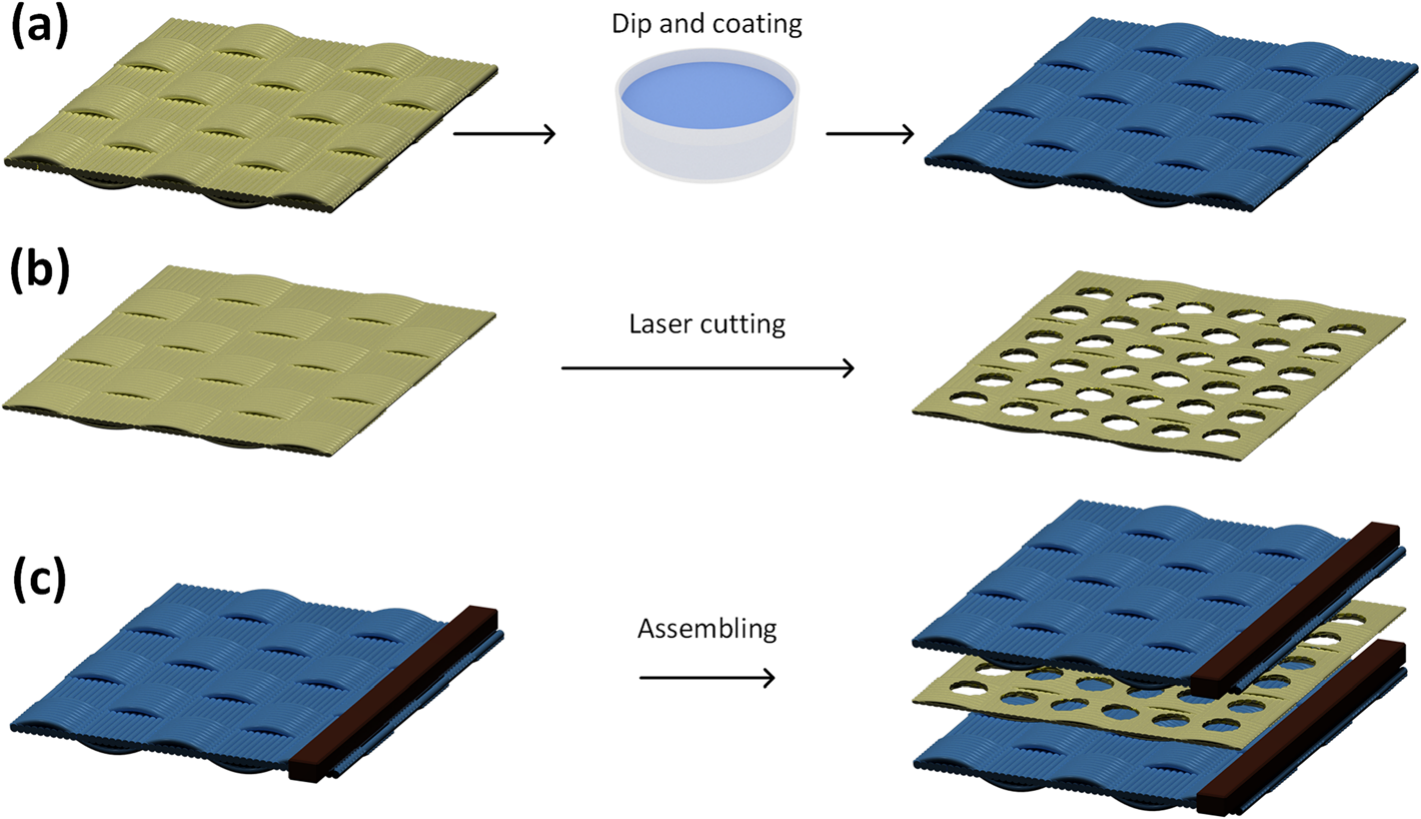
Fabrication process of the all-textile-based pressure sensor. **a** The preparation process of AgNW-coated cotton. **b** The fabrication process of mesh spacer cotton. **c** The assembling process of pressure sensor

The all-textile-based pressure sensor was fabricated using the “dipping and drying” process [31] (Fig. 1). First, the cotton fabrics were cleaned with DI and anhydrous ethanol each for 15 min. Second, the fabrics were dipped into the obtained solution of AgNWs for 20 min and followed by drying at 90 °C for 10 min (Fig. 1a). Then, the copper electrodes were attached to the surface of the AgNW-coated fabrics with silver paste and dried at 90 °C for 1 h. Meanwhile, the cotton mesh spacers with different hole diameters were fabricated by a laser etcher process (Fig. 1b). Finally, the double-layered sensing fabric with an inserted cotton mesh spacer was assembled by a face-to-face package process (Fig. 1c).

### Characterization

The scanning electron microscopy (SEM) images of the AgNW-coated fabric surfaces were taken via a GeminiSEM 500 (ZEISS, New York, America) at 5 kV. The current response of the pressure sensors was recorded using a digital source meter (Keithley 4200, America) and measured using a digital force gauge (SJS-500V, China).

## Results and Discussion

**Fig. 2:**
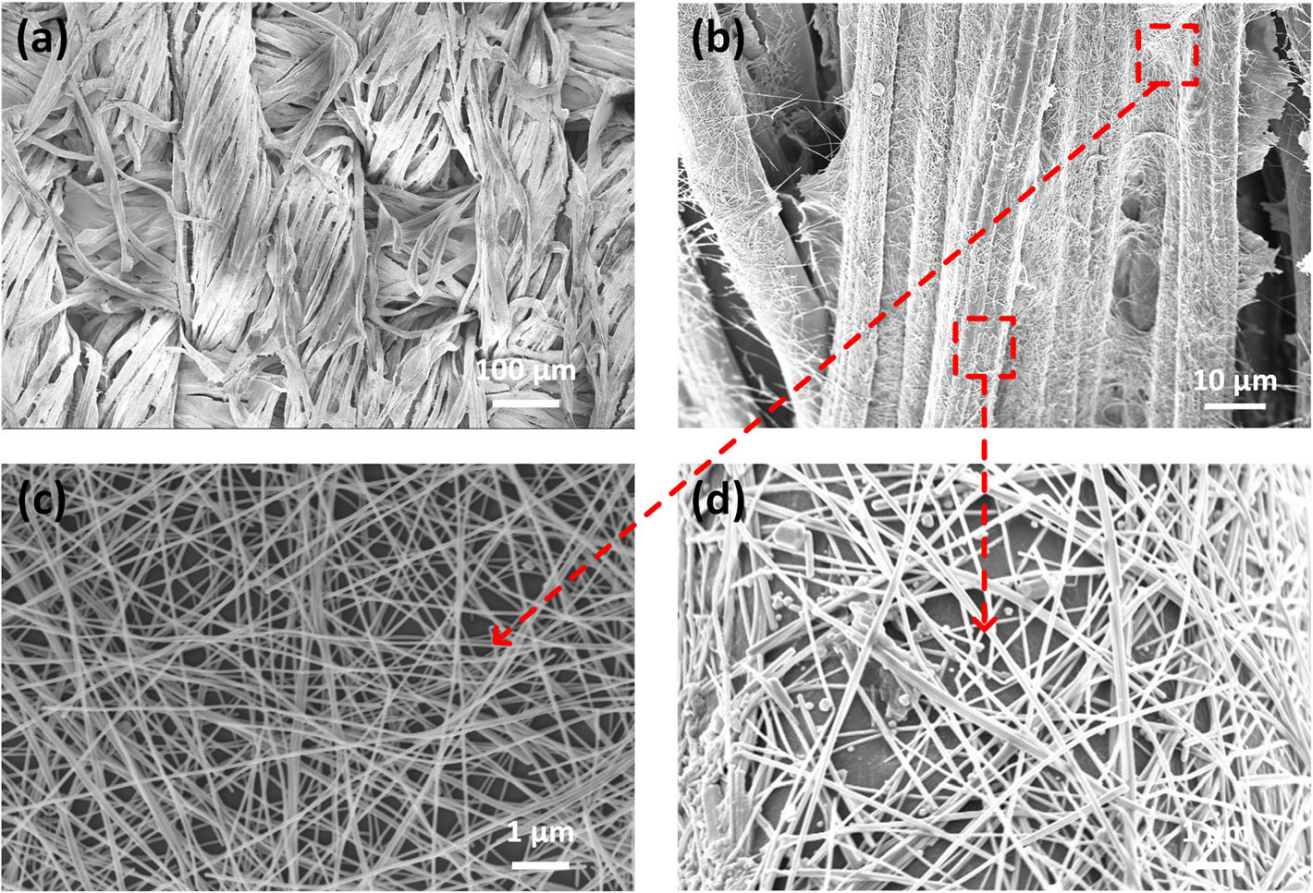
The morphology of the AgNWs-coated fabrics. **a**–**d** The SEM images of the surface morphology of AgNWs-coated fabric with different position of AgNWs and different magnification, in which **c** is the SEM image of the AgNWs between the yarns and **d** the AgNWs coated on the single fiber

Figure 2 shows SEM images of the morphology of the AgNWs-coated fabric with different magnification. As shown in Fig. 2a, the yarns of the cotton were layered naturally with porous structure. The outmost surface of the fabric is covered by AgNWs (Fig. 2b), on which nanowires are uniformly wrapped on the fibers. Particularly, between the neighboring yarns, there are empty spacings that are bridged by the attached AgNW conductive networks (Fig. 2c). To be noticed, long and uniform wires were observed between adjacent yarns, and the average diameter of AgNW is around 55 nm. In Fig. 2d, the AgNWs are homogenously formed at main area of the yarn surface, while disconnected at some point due to the poor adhesion. Furthermore, the distant between nanowires adhered on the individual yarn is relatively larger than that of the nanowires between neighboring yarns.

Also, the density of the AgNWs on the surface of the fabrics was adjusted by the times of dip-coating cycles. The AgNWs-coated fabric with 1 dip cycle and 5 dip cycles was shown in Fig. S1 and Fig. 2d, respectively. Compared with the high density one, the nanowire mesh spacings of 1 dip cycle were increased from below 1 to 2–4 μm.

The composition of the AgNWs-coated fabric was also investigated by the energy dispersive X-ray spectroscopy (EDS), as illustrated in the inset of Fig. S2. In addition to the C and O contents which mainly attributed from cotton, Ag element was also observed, indicating the distribution of AgNWs on the cotton.

**Fig. 3:**
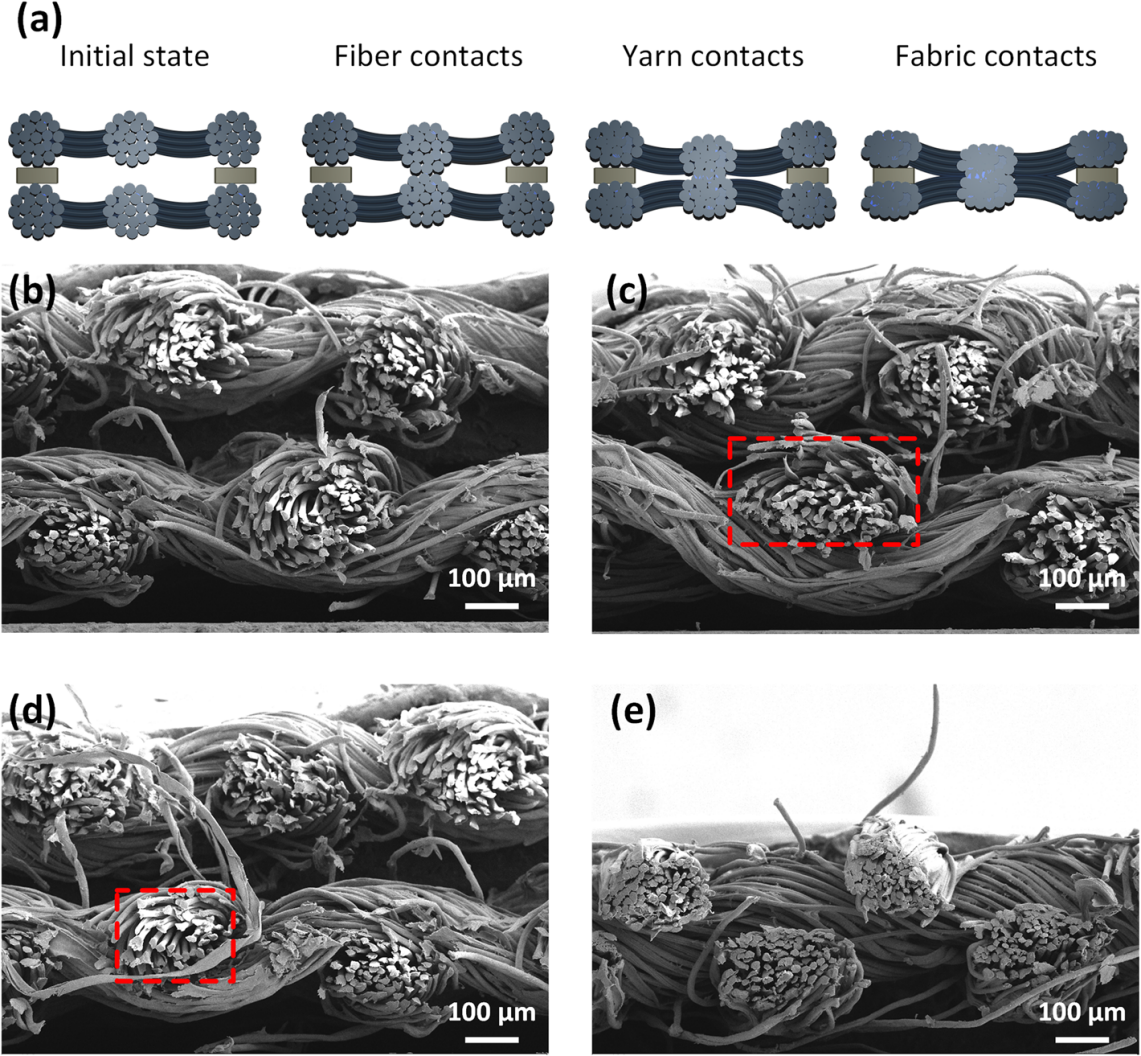
The sensing principle of pressure sensor. **a** Schematic illustration of pressure sensing. **b**–**e** The cross-section SEM images of the AgNWs-coated fabric under different pressure

The sensing principle of the pressure sensor is shown in Fig. 3a, and the cross-section SEM images of the sensor with different pressures are shown in Fig. 3b–e. In the unloading state, the initial resistance is large, which is caused by the non-contact AgNWs on the fabrics (Fig. 3b). Once the pressure was applied, the increasing fiber-scale contacts of nanowires on the adjacent fabrics contributed to a decrease in the resistance (Fig. 3c). Furthermore, when the nanowires on the fabrics were contacted completely, the continued pressure loaded on the fibers then increased the yarn-scale contacts. As Fig. 3d shown, the yarns length in *Y* direction are reduced from about 200 to 160 μm, confirming the compression between the yarns. According to the cross-section SEM images, the AgNWs were formed both on the yarn surface and inside the adjacent yarns (Fig. S3). When the pressure was applied and the yarns were compressed, the AgNWs inside the yarns could contact and further reduced the resistance of the sensor. With loading pressure increasing, the neighboring fabrics were compressed (Fig. 3e); fabric-scale contacts further reduced the resistance of the sensor due to the increased contact area between the facing fabrics. At this point, the total thickness of the double-layered fabrics was reduced from 600 to 350 μm. Therefore, the pressure sensing of the sensors was determined by the synergistic effect of the fiber/yarn/fabric multi-scale contact. These cross-section SEM images further confirmed the pressure sensing mechanism.

The influence of bending on the surface morphology of the AgNWs-coated fabrics was investigated by cross-section SEM images shown in Fig. S4. With the little bending deformation, there is no obvious crack and peeling off problem of AgNWs network on the fabrics (Fig. S4b) compared with initial state (Fig. S4a). In order to further investigate the influence of bending deformation, the SEM images of AgNWs-coated fabrics with 500 times bending cycles were taken and shown in Fig. S5. Fig S5 shows many delaminated spots which potentially occur device degradation. This result indicates that the stability of the AgNWs-coated fabric need to be further improved in the future.

**Fig. 4:**
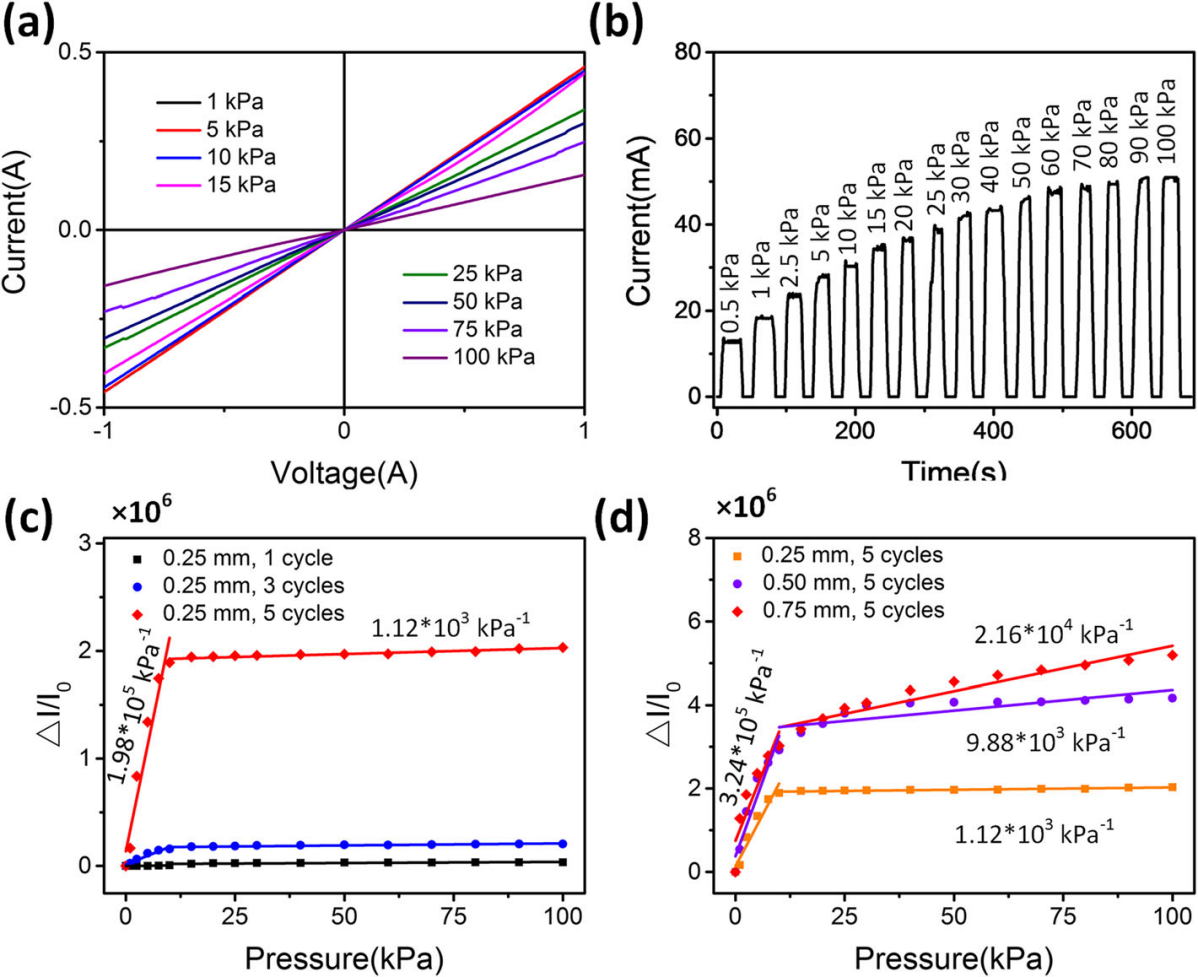
Performances of the pressure sensors. **a** I-V curves of the pressure sensor with different applied pressures. **b** The current response of the sensor under different pressures. **c**, **d** The performance comparison of the pressure sensors with different dip-coating cycles and mesh hole diameters

Fig. 4a shows the current-voltage curves of the pressure sensor under different pressures. When the applied pressure increased from 0 to 100 kPa, the resistance of the sensor was decreased. Furthermore, the response of sensor was steady and fell in line under Ohm’s law [32]. The current of pressure sensor is shown in Fig. 4b, which is relatively constant under different applied pressure, revealing that the response of the sensor is stable for different pressures. Therefore, the results provide excellent electrical stability for the potential application of the pressure sensor.

To investigate the performance of pressure sensors, the relative current changes (Δ*I*/*I*_0_) versus pressure with the different AgNWs dip-coating cycles and mesh hole diameters were shown in Fig. 4c, d. Here, the sensitivity of the pressure sensor was defined as *S* = (Δ*I*/*I*_0_)/*P*, where *P* denotes the applied pressure. At a mesh hole diameter of 0.25 mm, the sensitivity of the pressure sensor was strongly dependent on the AgNWs dip-coating cycles. The sensitivity of the sensors was improved from 2.12 × 10^3^ kPa^−1^ to 1.98 × 10^5^ kPa^−1^ within the range of 0–10 kPa when the dip-coating cycles increased from 1 to 5. In addition, the sensitivity improved from 764 to 1.12 × 10^3^ kPa^–1^ at 10–100 kPa. The improvement of sensitivity with high dip-coating cycles is mainly attributed to the increase of the AgNWs densities.

Furthermore, the dependence of hole diameter was subsequently characterized. The pressure sensors with 5 dip-coating cycles exhibited enhancing sensitivities with increasing diameters, which were increased from 1.12 × 10^3^, 9.88 × 10^3^, to 2.16 × 10^4^ kPa^–1^ within the pressure range of 10–100 kPa, respectively. The enhancement in the sensitivity was mainly attributed to the increased contact area through the larger holes. However, once the diameter exceeded 1 mm with 4 cycles, the initial interface of the facing fabrics resulted in more contact in the unloading state, thus, significantly lowering the contact resistance between the fabrics (Fig. S6). Furthermore, when the thickness of the spacer cotton is changed, the performances of the sensors get worse (Fig. S7). The sensor with lower thickness shows a decrease of ∆*I*/*I*_0_ due to the contacting of the facing fabrics in the initial state (Fig. S7a). In addition, higher thickness reduces the contact of the fabrics. When the thickness of spacer cotton increased to 1 mm, the AgNWs on the fabrics were not contact until the pressure exceed 10 kPa (Fig. S7c).

Notably, the pressure sensor clearly exhibited two linear current segments; the sensitivity increases sharply in the low-pressure range and increases gradually in the high-pressure range. According to the sensing principle as we mentioned above, in the low-pressure range, the adjacent AgNWs contact plays an important role in increased current. Furthermore, when the pressure is increased to 10–15 kPa, the AgNWs on the interface were contact completely. The current changes were mainly determined by the contact resistance between the yarns and fabrics, which was relatively stable. Contact between the yarn and fabric scales played a more significant role in the sensing mechanism at dip-coating cycles of 5 times and diameter of 0.75 mm by enhancing the sensitivity and linear range. Therefore, the diameter of 0.75 mm (Fig. 4d) exhibited a higher sensitivity and larger linear range due to increased contact [33, 34].

**Fig. 5:**
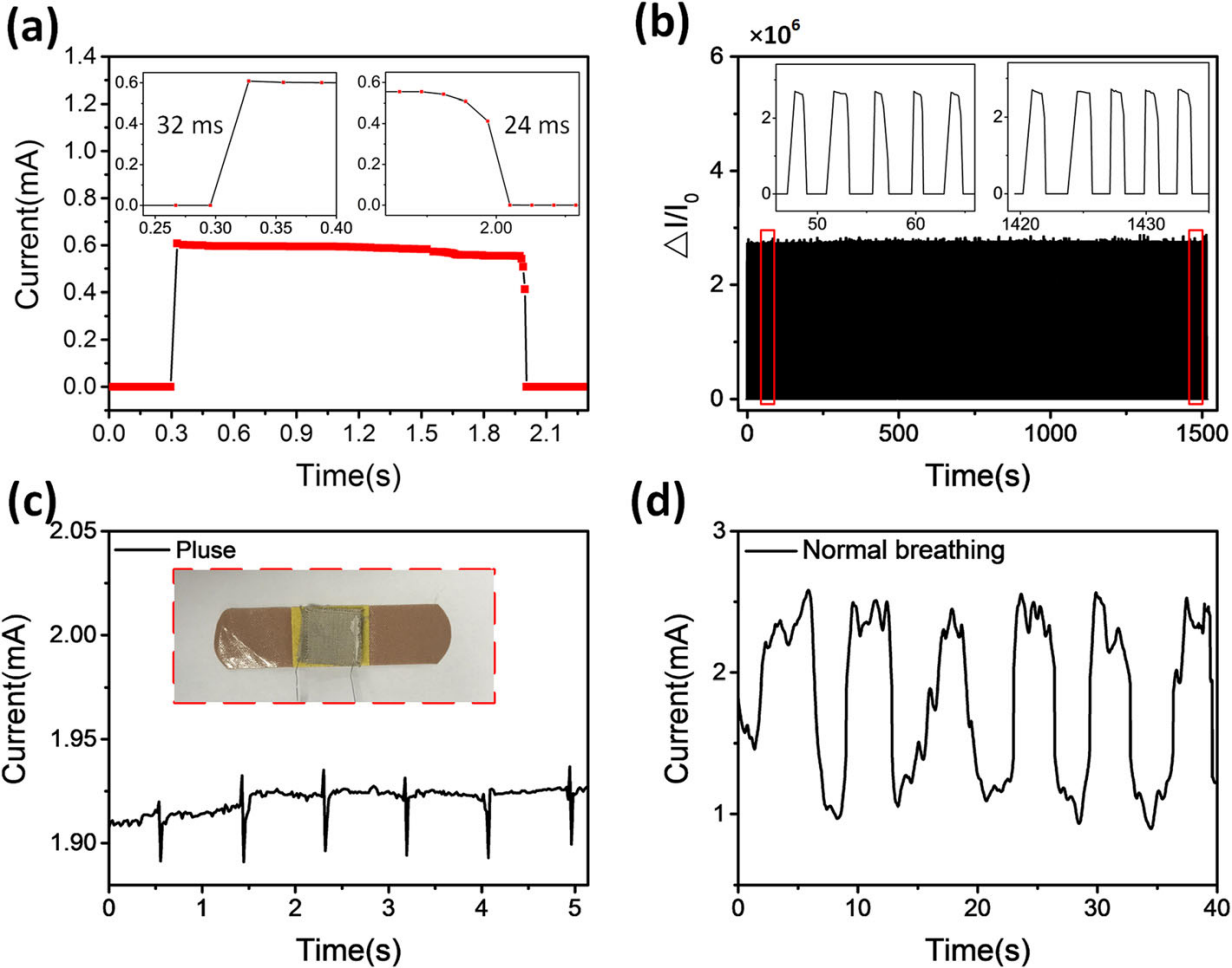
**a** Response/release times of the device. **b** The cycling test of the device under a pressure of 10 kPa. **c** Wrist pulse signal of the human user. **d** The current signal responds to respiration under normal breathing

The dynamic response of the device was studied under loading/unloading pressure cycles. The sensor exhibited an immediate response to the cyclic pressures. The time-resolved response was analyzed to quantify the response and relaxation times (Fig. 5a). The measured response and relaxation times were 32 and 24 ms, respectively. The performance of the sensor under difference pressure is also investigated and shown in Fig. S8. The sensor clearly distinguished a subtle pressure of 50 Pa, indicating the excellent performance of the sensor. The Δ*I*/*I*_0_ with an applied pressure of 10 kPa with 1000 loading cycles was used to verify the repeatability of the device (Fig. 5b). The results show the excellent stability of the pressure sensors. Furthermore, the air permeability of both normal cotton and AgNW-coated cotton was investigated. Despite the air permeability was reduced from 787.3 to 252.6 mm/s, this value is still much higher than the recently reported ones [35, 36]. This result demonstrated that the wearable pressure sensors based on silver nanowire-coated fabrics remain good air permeability because of its high porosity.

Due to the natural flexibility of fabrics and high sensitivity of sensors, the pressure sensor was wearable and able to detect mechanical signals such as physiological pulse and respiratory rate. First, the device was attached at the wrist using an adhesive bandage to monitor the pulse pressure. Fig. 5c presents the real-time recorded data, in which the pulse rates were measured to be ≈ 72 beats min^–1^. In addition, the sensor was also attached to a mask to detect respiration states. Fig. 5d indicates that the normal respiratory rate of 10 breaths per minute of an adult and a square-like wave for normal breathing. Furthermore, the width of the waveband indicated the maintained time of breathing. These results suggest that the pressure sensor with high sensitivity and superiority has great potential in wearable healthcare device applications.

## Conclusion

In this work, the AgNWs were fabricated by hydrothermal method, and the morphology was characterized and analyzed. An all-textile-based pressure sensor was fabricated by inserting a cotton mesh spacer between the double-layered AgNW-coated cottons. Owing to the collective effect of the fiber/yarn/fabric multi-scale contacts, the sensor has extremely high sensitivity (3.24 × 10^5^ kPa^−1^ at 0–10 kPa and 2.16 × 10^4^ kPa^−1^ at 10–100 kPa, respectively), fast response/recovery time (32/24 ms), high stability (1000 cycles), and wide pressure range (0–100 kPa). The physiological signals monitoring such as pulse pressure has been successfully demonstrated. With a facile and efficient method for fabrication, such an ultrasensitive pressure sensor will promote a wide application in next generation development of smart clothes, activity monitoring, and healthcare device.

## Supplementary information

**Additional file 1: Fig. S1** The SEM image of the fabric with 1 cycle dip-coated AgNWs that attached on a single yarn. **Fig. S2** The EDS analysis of the AgNWs-coated fabric. **Fig. S3** The morphology of the AgNWs-coated fabric. **a** The AgNWs coated on the surface of the yarns. **b** the AgNWs coated inside the yarns. **Fig. S4** The cross-section SEM images of AgNWs-coated fabric **a** before and **b** after bending. **Fig. S5** The morphology of the AgNWs-coated fabric after 500 times bending. **Fig. S6** The ∆I/I_0_ of the pressure sensors with a mesh hole diameters of 1 mm. **Fig. S7** The ∆I/I_0_ of the pressure sensors with different thickness of spacer cotton. **Fig. S8** The current of the pressure sensor under the pressure of 200 Pa

## Data Availability

The authors declare that the materials and data are available to the readers, and all conclusions made in this manuscript are based on the data which are all presented and shown in this paper.

## Abbreviations

CNTs: Carbon nanotubes
AgNW: Ag nanowire
PVP: polyvinyl pyrrolidone
EG: Ethylene glycol
NaCl: Sodium chloride
DI: Deionized water
SEM: Scanning electron microscope
EDS: Energy dispersive X-ray spectroscopy
