# Supplementary material for: Ultrasensitive Wearable Pressure Sensors Based on Silver Nanowire-Coated Fabrics
Source: Nanoscale Res Lett. 2020 Mar 30;15:70. doi: 10.1186/s11671-020-03303-2 (PMC7105525; doi:10.1186/s11671-020-03303-2)
Supplement: Supplementary file 1 — Additional file 1: Fig. S1 The SEM image of the fabric with 1 cycle dip-coated AgNWs that attached on a single yarn. Fig. S2 The EDS analysis of the AgNWs-coated fabric. Fig. S3 The morphology of the AgNWs-coated fabric. a The AgNWs coated on the surface of the yarns. b the AgNWs coated inside the yarns. Fig. S4 The cross-section SEM images of AgNWs-coated fabric a before and b after bending. Fig. S5 The morphology of the AgNWs-coated fabric after 500 times bending. Fig. S6 The ∆I/I0 of the pressure sensors with a mesh hole diameters of 1 mm. Fig. S7 The ∆I/I0 of the pressure sensors with different thickness of spacer cotton. Fig. S8 The current of the pressure sensor under the pressure of 200 Pa [file 11671_2020_3303_MOESM1_ESM.doc]

**Supporting Information**

Ultrasensitive wearable pressure sensors based on Silver nanowire-coated fabrics

Yunlu Lian, He Yu*, Mingyuan Wang, Xiaonan Yang, Hefei Zhang

State Key Laboratory of Electronic Thin Films and Integrated Devices, University of Electronic Science and Technology of China (UESTC), 610054, P. R. China.

(E-mail: yuhe@uestc.edu.cn)


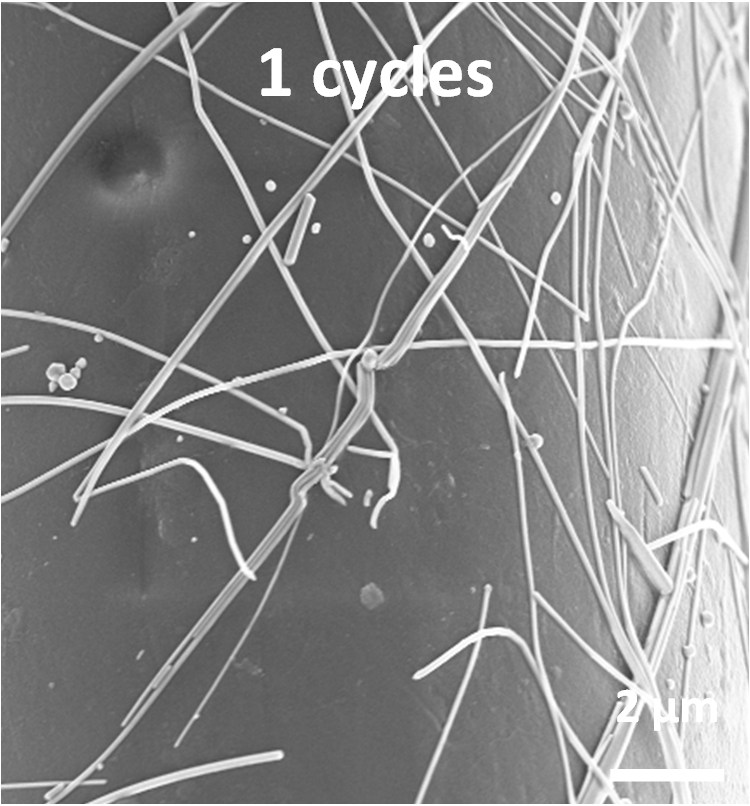


**Fig. S1** The SEM image of the fabric with 1 cycle dip-coated AgNWs that attached on a single yarn


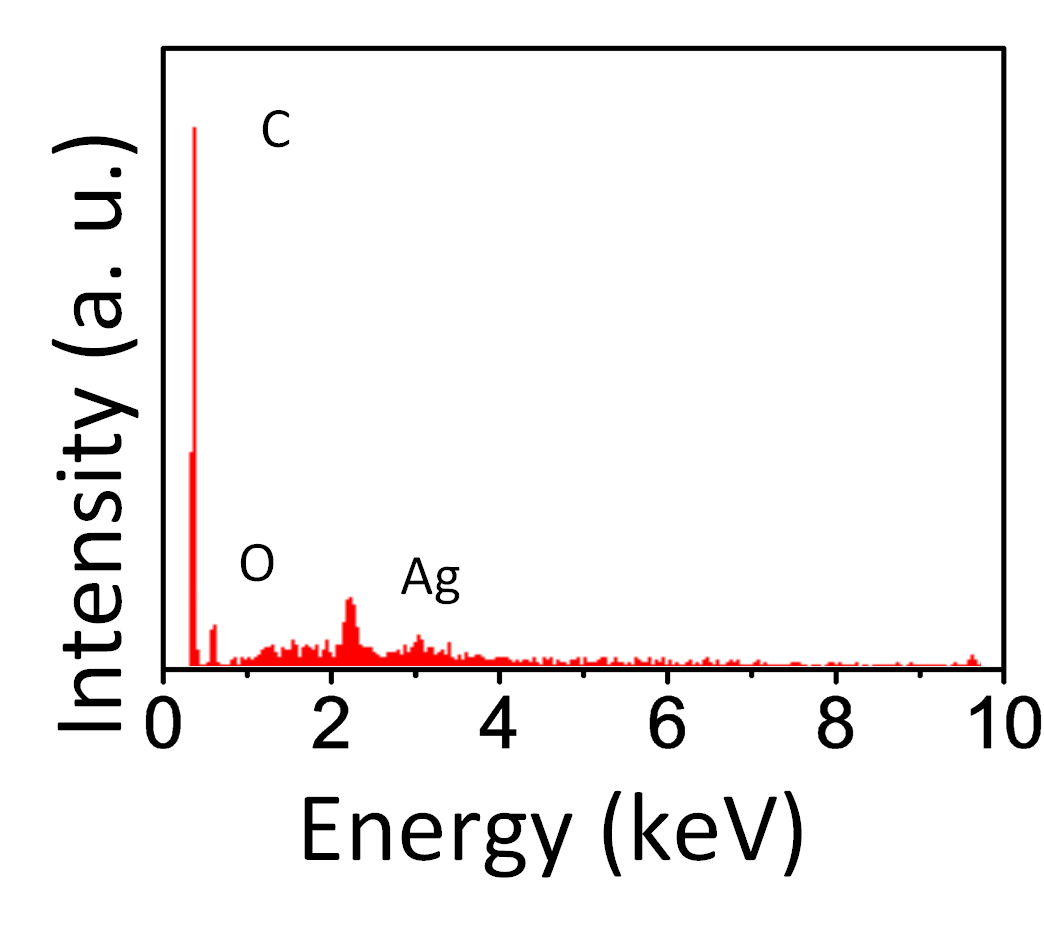


**Fig. S2** The EDS analysis of the AgNWs-coated fabric


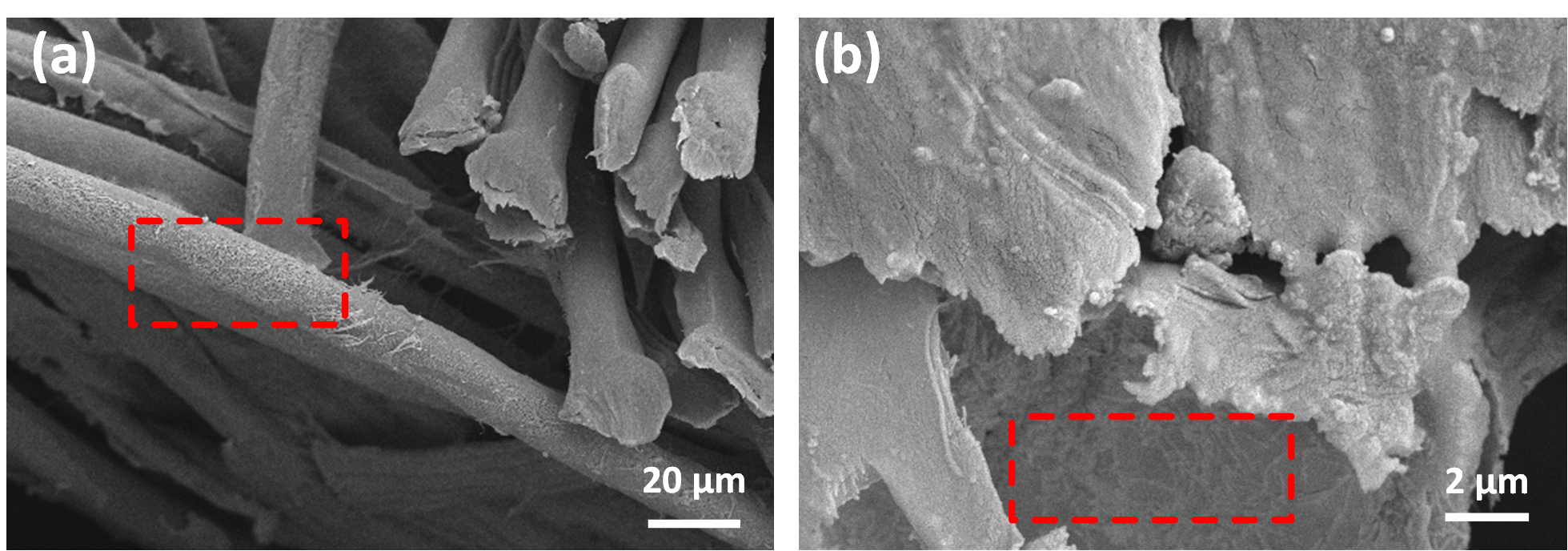


**Fig. S3** The morphology of the AgNWs-coated fabric. **a** The AgNWs coated on the surface of the yarns. **b** the AgNWs coated inside the yarns


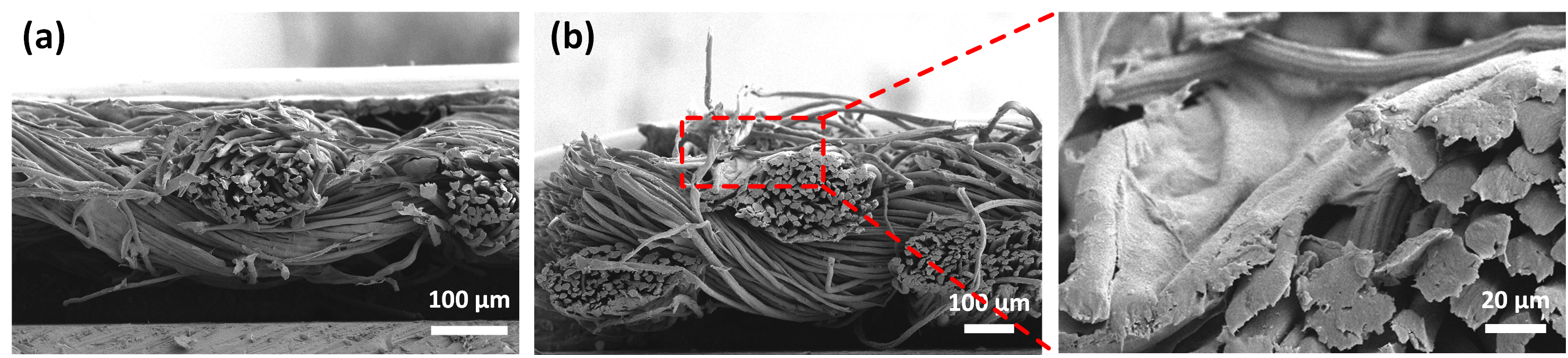


**Fig. S4** The cross-section SEM images of AgNWs-coated fabric **a** before and **b** after bending


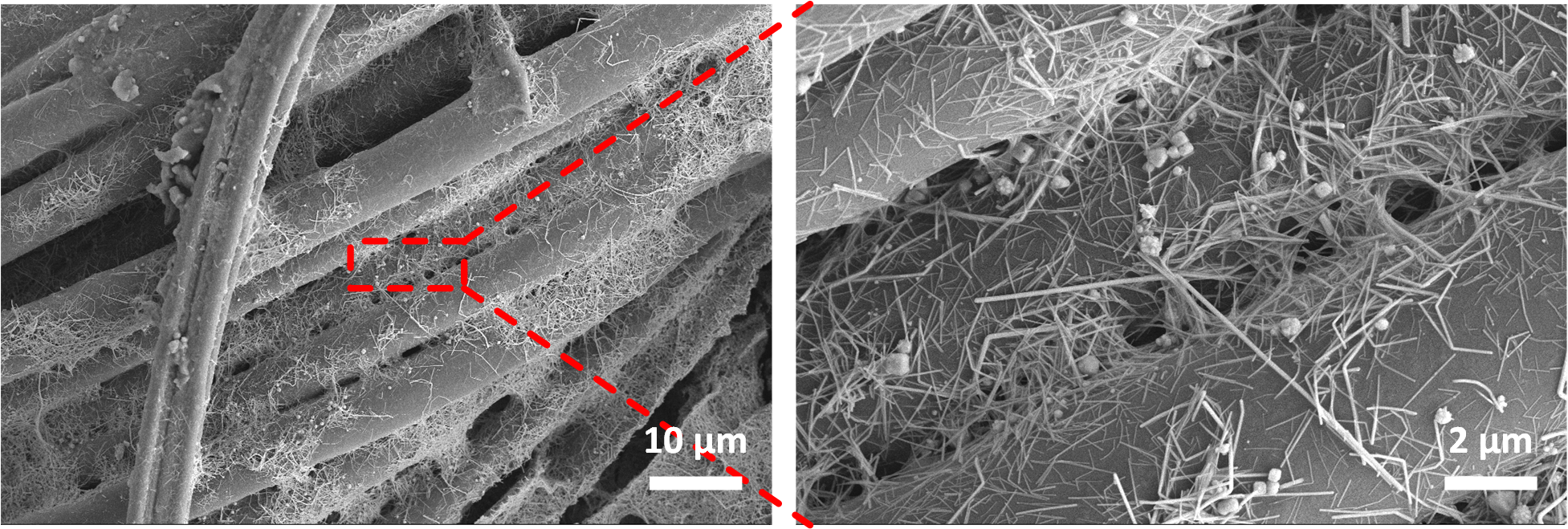


**Fig. S5** The morphology of the AgNWs-coated fabric after 500 times bending


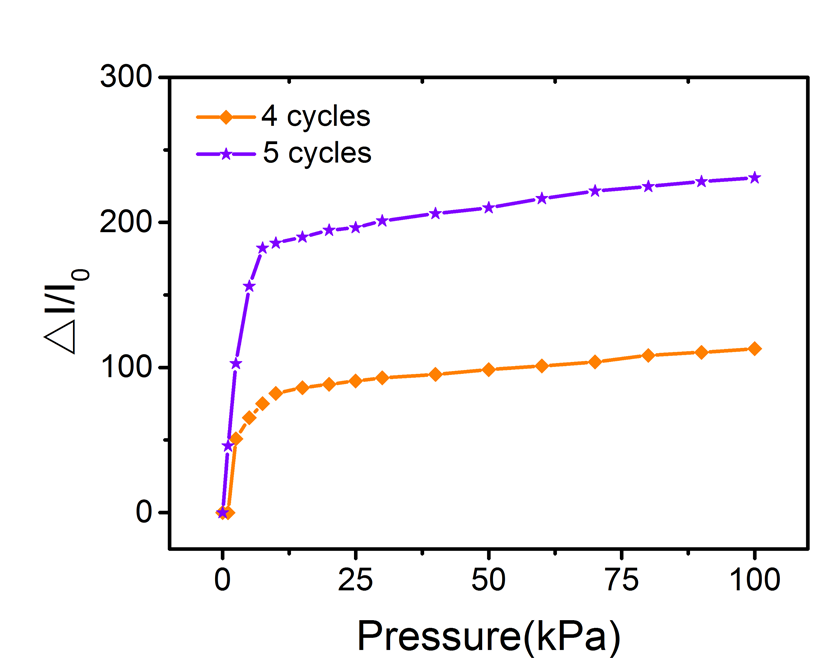


**Fig. S6** The ∆I/I_0_ of the pressure sensors with a mesh hole diameters of 1 mm


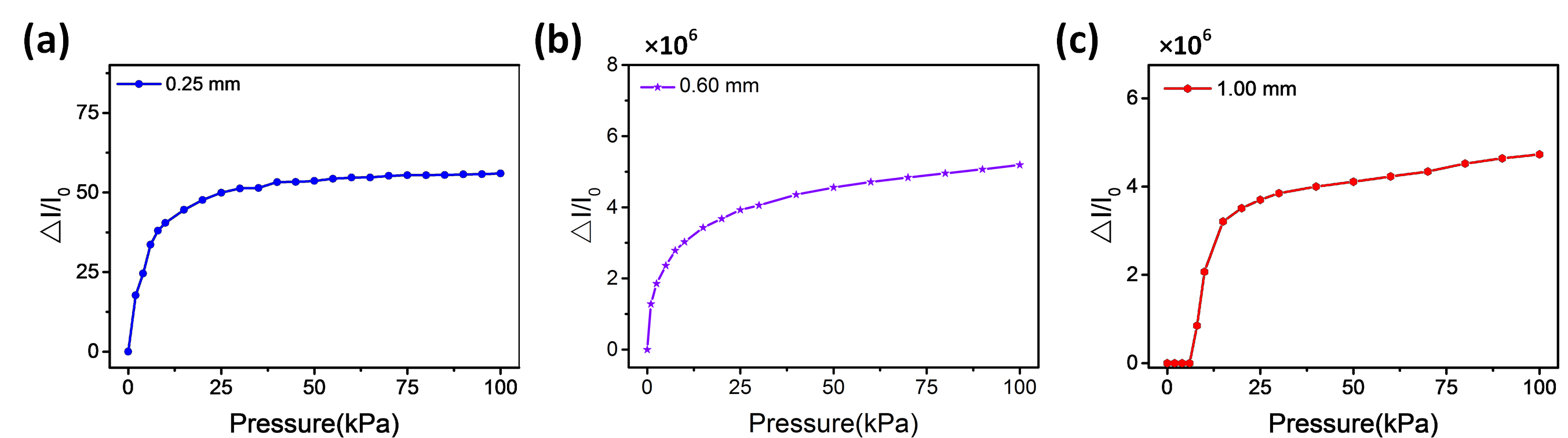


**Fig. S7** The ∆I/I_0_ of the pressure sensors with different thickness of spacer cotton


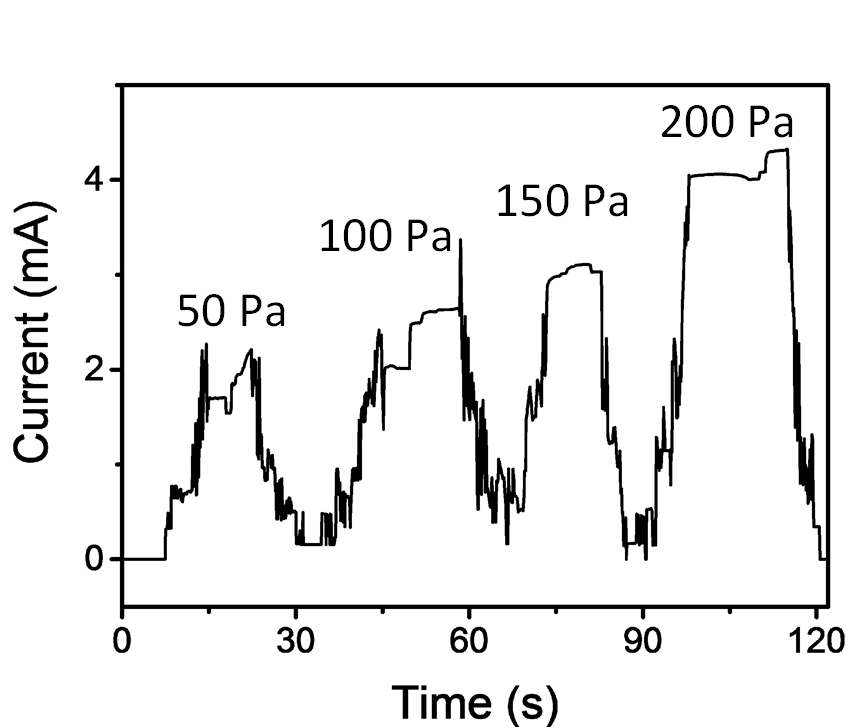


**Fig. S8** The current of the pressure sensor under the pressure of 200 Pa
